# Quality of Vegetables Based on Total Phenolic Concentration Is Lower in More Rural Consumer Food Environments in a Rural American State

**DOI:** 10.3390/ijerph14080924

**Published:** 2017-08-17

**Authors:** Selena Ahmed, Carmen Byker Shanks

**Affiliations:** Food and Health Lab at Montana State University, Sustainable Food and Bioenergy Systems Program, Department of Health and Human Development, Montana State University, Bozeman, MT 59717, USA; cbykershanks@montana.edu

**Keywords:** food environments, vegetables, phytochemicals, rural, nutrition, food quality

## Abstract

While daily consumption of fruits and vegetables (FVs) is widely recognized to be associated with supporting nutrition and health, disparities exist in consumer food environments regarding access to high-quality produce based on location. The purpose of this study was to evaluate FV quality using total phenolic (TP) scores (a phytochemical measure for health-promoting attributes, flavor, appearance, and shelf-life) in consumer food environments along a rural to urban continuum in the rural state of Montana, United States. Significant differences were found in the means of the FV TP scores (*p* < 0.0001) and vegetable TP scores (*p* < 0.0001) on the basis of rurality, while no significant difference was found for fruit TP scores by rurality (*p* < 0.2158). Specifically, FV TP scores and vegetable TP scores were highest for the least rural stores and lowest for the most rural stores. Results indicate an access gap to high-quality vegetables in more rural and more health-disparate consumer food environments of Montana compared to urban food environments. Findings highlight that food and nutrition interventions should aim to increase vegetable quality in rural consumer food environments in the state of Montana towards enhancing dietary quality and food choices. Future studies are called for that examine TP scores of a wide range of FVs in diverse food environments globally. Studies are further needed that examine linkages between FV quality, food choices, diets, and health outcomes towards enhancing food environments for public health.

## 1. Introduction

The daily consumption of FVs is integral for human nutrition and is associated with reduced risk of obesity and diet-related chronic disease, including heart disease, cancer, and diabetes [1,2]. Diet-related chronic disease is one of the major types of non-communicable diseases that are growing globally including in both developed and developing countries [3]. However, the majority of adults in developed and developing countries consume fewer FVs than the amounts recommended by dietary guidelines [4,5]. Consumption of FVs is dependent on multiple socio-ecological factors, including personal preferences and other individual characteristics, social and cultural norms linked to lifestyles and belief systems, and offerings in the consumer-built food environment [6]. The consumer food environment refers to the spaces and products that are created by people for the provision of food, including supermarkets, restaurants, and institutions [7]. Previous research has identified the food environment as a determinant of health by directly influencing the availability, affordability, accessibility, and desirability of foods that impacts dietary choices and, ultimately, nutrition and health [8].

A vast body of literature highlights that disparities exist in accessing healthy foods, including FVs in the consumer food environment depending on rural versus urban locations [6,9,10]. Specifically, rural adult populations in the United States have been shown to have less access to FVs in the consumer food environment compared to those in urban areas [6]. Reduced access to FVs in the consumer food environment is recognized to contribute to disparities in produce consumption and dietary intake between rural and urban communities [9]. Previous studies have demonstrated that adults in rural locations consume fewer FVs compared to adults in urban areas [9]. These disparities in FV consumption between rural and urban communities potentially have notable implications for health outcomes including diet-related chronic disease [10].

In addition to variations in overall access to specific FVs between urban and rural locations, consumers located in different food environments also have variable access to differing quality of produce [6]. The quality of FVs is determined by a range of characteristics including organoleptic properties (such as taste, aroma, and texture), shelf life, and the presence and concentrations of phytochemicals (i.e., bioactive food components) that are associated with nutrition and health attributes of produce [11]. Produce quality, including its nutritional and health properties, as well as sensory attributes, may have a mediating effect on consumer purchasing decisions [8,12].

The key factors that contribute to the nutritional and health benefits of FVs are the presence of vitamins and dietary phytochemicals that influence produce quality [12]. Dietary phytochemicals are non-nutrient constituents of plant-based foods that support human nutrition and health by mitigating micronutrient deficiencies [13] and the risk of cancer, heart disease, and diabetes [12]. There are over 5000 phytochemicals identified to date that can be grouped into the following five classes: (1) phenolics, (2) carotenoids and other terpenes, (3) alkaloids and other nitrogen-containing compounds, (4) phytosterols and, (5) organosulfur compounds [12]. Phenolics and carotenoids are the two most studied and abundant groups of dietary phytochemicals associated with antioxidant properties and other mechanisms for mitigating diet-related chronic disease and micronutrient deficiencies [12,13,14]. In addition, the phenolic class of phytochemicals has been identified to play an important role in FV quality through an influence on flavor, appearance, health-promoting attributes, and stability [15]. Examples of phenolic phytochemicals are the anthocyanins that contribute to the blue and purple color of blueberries and grapes [16]. Phenolic phytochemicals have been identified in commonly consumed fruits [17,18] and vegetables [19,20,21].

The type of phenolic compounds and the concentration of specific phytochemicals notably vary between and within different FVs and type of plant tissue [11]. Phenolic compounds play a role in secondary metabolism in plants, including for defense against predators, pathogens, and abiotic stress [11]. Levels of phenolic concentrations in FVs vary based on a range of genetic, environmental, processing, and storage factors involved in cultivation and distribution [11]. While it is recognized that phenolic concentrations of FVs vary with production and distribution variables, little is known about how FV quality based on phenolic concentrations varies with the location in the consumer food environment beyond observational data [22]. The majority of food environment studies in the past two decades have focused on evaluating variability in the availability and price of foods across several food groups based on geographic factors such as rurality and density of vendors [23,24].

The purpose of this study was to evaluate FV quality using total phenolic (TP) concentration scores as a measure of health-promoting attributes, flavor, appearance, and shelf-life of produce in rural and urban built food environments in the rural state of Montana, United States towards elucidating access gaps to high-quality produce based on location. We compared how TP scores vary with rurality as classified by United States Department of Agriculture (USDA) rural-urban continuum codes (RUCC) [25] as well as with produce quality scores derived from the Nutrition Environment Measurement Survey for Stores (NEMS-S) [26], the most widely used food environment survey tool in the United States. NEMS-S takes into consideration FV quality through a dichotomous evaluation where the survey investigator records if 50% or more of the specific FV assessed is considered acceptable. Previous work by this study’s authors demonstrated that NEMS-S quality scores varied by rurality based on RUCC with the least rural area having the highest NEMS-S quality scores [6]. The present study builds on this previous work by this study’s authors on examining FV quality in the rural state of Montana in the United States. This is the first study to our knowledge that has compared FV quality using phytochemical measures between consumer food environments along a rural to urban continuum. It is expected that our methodological approach of measuring total phenolic (TP) scores to assess FV quality in the consumer food environment can be used by other scientists and practitioners in order to enhance the understanding of food environments and to help elucidate socio-ecological determinants of health in the food system. Findings from this study and future related studies that adapt our methodological approach can be applied for informing the design of food and nutrition interventions towards preventing diet-related chronic disease and health disparities on the basis of the rurality of location.

## 2. Materials and Methods

### 2.1. Collection of FVs

FVs (*n* = 324) were collected from 12 grocery stores in 11 urban and rural communities in the rural state of Montana in the United States. Stores were randomly selected based on the 2013 United States Department of Agriculture rural-urban continuum codes (RUCC) classifications that designate metropolitan (urban) counties by the population size of their metro area, and nonmetropolitan (rural) counties by degree of urbanization and adjacency to a metro area [25]. Rural-urban continuum codes from 1 through 3 are classified as metro (urban) and 4 through 10 are classified as non-metro (rural). The sample size of counties included in this study was determined as twenty percent of the rural (*n* = 10) and urban (*n* = 1) counties in the state of Montana that has a total of five metro counties and 51 non-metro counties. Thus, this study randomly selected ten rural counties and one urban county using a random number generator from a master list of counties and associated RUCC in Montana. The largest grocery store within the largest town of each county was selected for inclusion in this study for FV collections. If two grocery stores within a selected town were of near-equal size, both grocery stores were surveyed. The rationale for selected the largest grocery store in each surveyed town is based on observational pilot work that indicates this is where the consumer has access to the greatest variety of FVs and also where the majority of consumers within the town purchase food. This protocol resulted in sampling two stores with a RUCC of 3, one store with a RUCC of 6, two stores with a RUCC of 7, four stores with a RUCC of 8, and three stores with a RUCC of 9. This produce observational study was exempt from institutional review board review.

During sampling, store managers were asked regarding the origin and distribution of the sampled FVs. The sampled FVs had the same origin and distributor. However, the frequency of distribution and deliver of FVs varied based on location with the urban location having the greatest frequency of deliveries.

We assessed the first four types of fruits (banana, red delicious apple, navel oranges, red seedless grape), and the first five types of vegetables (carrot, tomato, green sweet bell pepper, broccoli, green leaf lettuce) listed in the NEMS-S produce quality assessment that represent the most consumed produce in the United States. Researchers randomly selected three of each produce type from the top, middle, and bottom of each produce display for a total target sample size of 324 individual FVs. FVs were placed on ice upon collection and transported to the investigators’ lab where they were frozen at −80° F until analysis.

### 2.2. Phytochemical Analysis and Scores

Fruits and vegetables (FVs) were processed and analyzed for total phenolic (TP) concentration using the spectrophotometric Folin-Ciocalteau reagent method as previously described [27]. Samples were analyzed in triplicate. Absorbance values were measured at 765 nm using a BioTek Synergy HT Microplate Reader (Winooski, VT, USA) using Gen5 2.05 software (BioTek, Winooski, VT, USA) and the results are expressed as gallic acid equivalents (GAE) in mg·g^−1^ dry plant material derived from a standard curve of GA concentration versus absorbance between 31.25 and 500 g·mL^−1^.

### 2.3. NEMS-S

The Nutrition Environment Measurement Survey for Stores (NEMS-S) was used to assess the observational quality of selected FVs in stores included in this study as described by Glanz et al. [26] at the same time the FVs were collected for this study and were previously reported on by this study’s authors [6]. As previously reported, the average NEMS-S total availability score was 17.6 (SD = 5.3; out of 30 possible points), the average total price score was 2.9 (SD = 3.0; out of −9 to 18 possible range), and the average total quality score (acceptability of FV) was 4.2 (SD = 1.9; out of six possible points). Overall, the average total NEMS-S score for the sampled stores was 24.7 (SD = 7.2; out of 54 possible points).

### 2.4. Statistical Analysis

Model fitting using a standard least square (LS) means function and analysis of variance was performed using JMP 12.0 (SAS Institute Inc., Cary, NC, USA) to determine how TP concentrations vary by USDA rural-urban continuum codes (RUCC). Fruit and vegetable (FV) TP scores were calculated based on LS means of TP concentrations of all analyzed FVs. Fruit TP scores and vegetable TP scores were calculated as the LS means of TP concentrations of analyzed FVs accordingly. The higher the value of the resulting TP scores for a given location, the higher the quality of FVs at that location. TP scores were plotted against RUCC values. A multiple comparison using the LS means Tukey Honest Significant Difference (HSD) test was applied to examine if TP scores significantly varied along a rural to urban continuum based on RUCC. Finally, we applied bivariate fit analyses to evaluate the relationship of FV TP scores by NEMS-S total scores and NEMS-S total quality scores. The significance level was set at *p* < 0.05.

## 3. Results

**Figure 1:**
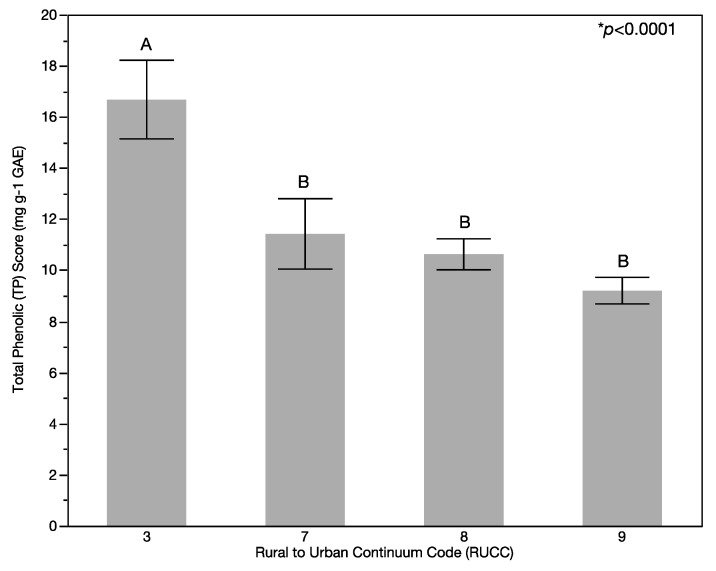
Variation of combined fruit and vegetable (FV) quality based on FV total phenolic (TP) scores with rurality. FV quality as determined by FV total phenolic (TP) scores is lower in more rural areas (higher rural urban continuum codes (RUCC) represent more rural areas). RUCC codes that have the same letter (A or B) above the standard error bar in the graph show no statistical difference while RUCC codes that have different letters above the bar in the graph are statistically different. Each error bar is constructed using one standard error from the mean.

**Figure 2:**
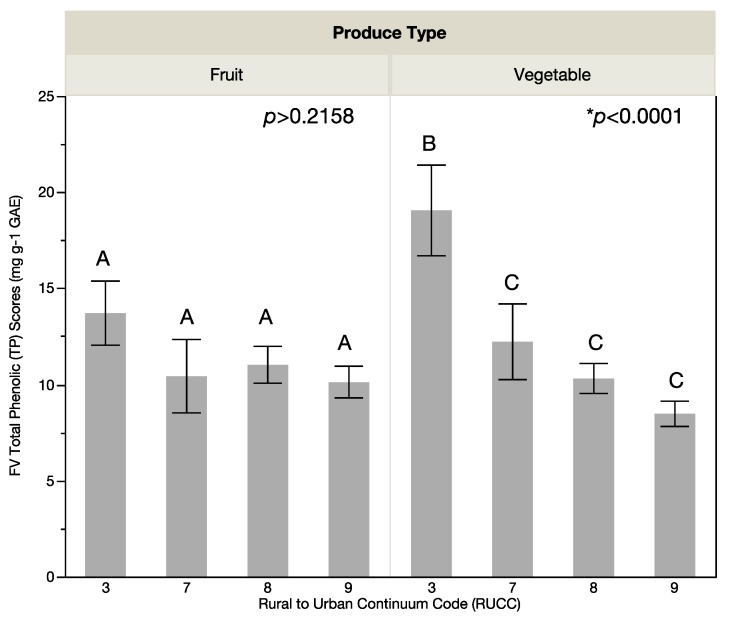
Variation of fruit quality and vegetable quality based total phenolic (TP) scores with rurality. No significant difference was found in fruit quality based on TP scores with rurality. Vegetable quality as determined by vegetable total phenolic (TP) scores is lower in more rural areas (higher rural urban continuum codes (RUCC) represent more rural areas). RUCC codes that have the same letter (A or B) above the standard error bar in the graph show no statistical difference while RUCC codes that have different letters above the bar in the graph are statistically different. Each error bar is constructed using one standard error from the mean.

Significant differences were found in overall produce quality as measured by total phenolic (TP) phytochemical scores on the basis of location. Specifically, significant differences were found in the means of the fruit and vegetable (FV) TP scores (*p* < 0.0001; Figure 1) and vegetable TP scores (*p* < 0.0001; Figure 2) on the basis of RUCC while no significant difference (*p* > 0.2158) was found for fruit TP scores (Figure 2) by USDA rural-urban continuum codes (RUCC). FV TP scores (Figure 1) and vegetable TP scores (Figure 2) were highest for the least rural stores (RUCC of 3) and lowest for the most rural stores (RUCC of 9). The LS means Tukey HSD multiple comparison analysis found significant differences in the total TP scores and the vegetable TP scores between RUCC 3 compared with RUCC 7, 8, and 9, while no significant differences were found for fruit TP quality scores. The mean of the FV TP scores was 11.17 mg·g^−1^ gallic acid equivalents (GAE) with a mean range of 9.20 mg·g^−1^ GAE (standard deviation (Std Dev) 6.05 mg·g^−1^ GAE) for the lowest scored RUCC (RUCC of 9) to a mean of 16.67 mg·g^−1^ GAE (Std Dev of 11.29 mg·g^−1^ GAE) for the highest scored RUCC (RUCC of 3). The mean vegetable TP score was 11.31 mg·g^−1^ GAE with a mean range from 8.47 mg·g^−1^ GAE (Std Dev 5.69 mg·g^−1^ GAE) for the lowest scored RUCC (RUCC of 9) to 19.05 mg·g^−1^ GAE (Std Dev 12.91 mg·g^−1^ GAE) for the highest scored RUCC (RUCC of 3). The mean of the fruit TP score was 10.99 mg·g^−1^ GAE with a mean range from 10.12 mg·g^−1^ GAE (Std Dev of 6.41 mg·g^−1^ GAE) for the lowest scored RUCC (RUCC of 9) to 13.71 mg·g^−1^ GAE for the highest scored RUCC (Std Dev of 8.18 mg·g^−1^ GAE). Significant differences were found in TP scores between types of vegetables (*p* < 0.0001) and types of fruit (*p* < 0.0001). For the vegetables sampled, peppers had the highest TP scores. For the fruit sampled, apples and oranges had the highest TP scores.

**Figure 3:**
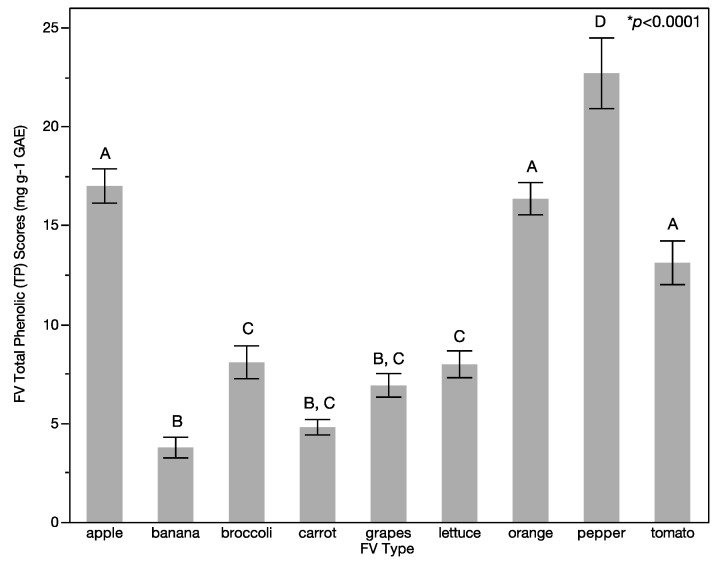
Variation of total phenolic (TP) scores by fruit and vegetable (FV) type. Significant difference was found in TP scores FV type. Specific FVs that have the same letter (A or B) above the bar in the graph show no statistical difference while RUCC codes that have different letters above the bar in the graph are statistically different. Each error bar is constructed using one standard error from the mean.

Comparison between types of FV found significant differences in TP scores (*p* < 0.0001; Figure 3). Specifically, peppers had the greatest TP scores (22.67 mg·g^−1^ GAE; Std Dev 10.70 mg·g^−1^ GAE) and bananas had the lowest TP scores (3.76 mg·g^−1^ GAE; Std Dev 3.13 mg·g^−1^ GAE). Peppers also had the greatest variability in TP scores as evaluated based on standard deviation of TP scores (Std Dev 10.70 mg·g^−1^ GAE) while carrots had the least variability of TP scores (Std Dev 2.32 mg·g^−1^ GAE).

Comparison of TP scores with scores with scores from the Nutrition Environment Measurement Survey for Stores (NEMS-S) demonstrated that higher vegetable TP scores were significantly correlated with higher NEMS-S total points and NEMS-S availability points (*p* < 0.006). No significant correlation was found with NEMS-S total quality points and NEMS-S total price points. Further, there were no significant correlations found between fruit TP scores and NEMS-S total points, NEMS-S availability points, NEMS-S total quality points and NEMS-S total price points.

## 4. Discussion

This study highlights that individuals in the rural consumer food environment in the American state of Montana have lower access compared to individuals in urban areas to high-quality vegetables on the basis of phenolic phytochemicals that influence health-promoting attributes, flavor, appearance, and shelf-life of produce. To our knowledge, this is the first study to compare FV quality using phytochemical measures between consumer food environments along a rural to urban continuum. It is expected that the methodological approach that we present of measuring total phenolic (TP) scores to assess FV quality in the consumer food environment can be used by other scientists and practitioners with the goal to improve food environments for public health. From a public health perspective, the lower vegetable quality in more rural areas is noteworthy as produce quality is a key determinant of FV consumption [28], as well as being linked to mitigating micronutrient deficiencies and chronic disease. Findings are in agreement with previous studies regarding the lower quality of vegetables based on appearance in more rural areas in the United States that are also associated with lower food access overall [6,29,30]. In addition to contributing a methodological approach to characterizing the food environment, findings contribute to the body of literature on the consumer environment as an important and modifiable determinant of health and wellbeing [7]. Results from this study and future related studies on characterizing FV quality in the consumer food environment can be applied to inform the design of food and nutrition interventions that support public health.

Limits to accessing high-quality produce in the food environment may foster poor food choices by rural consumers by driving consumers away from the produce section of the supermarket to the more heavily processed food aisles that have numerous products high in added sugars and low in nutrients. While this study did not evaluate linkages of FV quality to consumer food choices, FV consumption, and health outcomes, rural residents are recognized to consume fewer fruits and vegetables daily compared to more urban residents [9]. In addition, rural residents in the United States have higher risk for diet-related chronic disease compared to urban populations [31]. In the state of Montana, adults consume a daily median of 2.6 FV servings which is in contrast to the 5 FV daily servings as recommended by the USDA Dietary Guidelines for Americans 2015. This situation is starker in more rural areas of Montana that likely have lower rates of FV consumption and have higher rates of type 2 diabetes [17]. Additionally, lower income in more rural areas of Montana may further exacerbate the situation and lead to additional health disparities and marginalization of rural areas.

The lower vegetable phytochemical quality scores in more rural stores is likely due to limited food distribution and storage infrastructure (e.g., roads, frequency of delivery, storage technology) as we identified that the majority of sampled FVs had the same distributor while varying in the frequency of distribution. Phenolic phytochemicals in FVs notably degrade and decrease based on storage conditions and duration, among other factors [32]. This study points to the need for interventions addressing diet-related chronic disease to target the consumer environment in addition to lifestyle and behavioral factors [7]. In our case study site in the state of Montana, food and nutrition interventions should increase vegetable quality in rural consumer food environments by improving the storage and distribution infrastructure that supports access to high-quality produce towards preventing diet-related chronic disease and reducing health disparities on the basis of place.

While we carried out this study on total phenolic concentrations of FVs in the built food environment in the rural state of Montana, United States, it is expected that this approach can be used in varied communities globally, including low-income countries. Future research is called for to measure FV quality based on total phenolic concentrations in food environments around the world including in low-income, moderate-income, and high-income countries. Additionally, this study only evaluated FV quality in the largest food store in each town in the study area; research is called for to examine variations in FV quality in a range of store types and sizes within a community. Furthermore, future research is needed to implement the approach presented in this study using a diversity of FVs, as well as phytochemical classes and nutrients that were not included here. Lastly, future research is needed that measures the relationship of FV quality in the food environment based on total phenolic concentrations with food choices, dietary quality, and health outcomes in order to foster healthy food environments that support public health.

## 5. Conclusions

The consumer food environment is a determinant of health by directly influencing the availability, affordability, accessibility, and desirability of foods that ultimately influence dietary choices, nutrition, and health. In addition to access to specific foods, such as fruits and vegetables (FVs), the consumer food environments may also have variable access to differing quality of foods. FV quality is determined by a range of parameters including organoleptic properties (i.e., taste, aroma, and texture), shelf life, and the presence and concentrations of phytochemicals that are associated with nutrition and health attributes of produce. The quality of FVs may have a mediating effect on consumer purchasing decisions and ultimately impact human nutrition and health. This study provides evidence that FV quality based on total phenolic (TP) concentrations is lower in more rural areas of a rural American state where there is documented heightened diet-related chronic disease and health outcomes. Specifically, significant differences were found in the means of the FV TP scores and vegetable TP scores on the basis of rurality, while no significant difference was found for fruit TP scores by rurality. Further, FV TP scores and vegetable TP scores were highest for the least rural stores and lowest for the most rural stores. In addition, this study found that higher vegetable TP scores were significantly correlated with higher NEMS-S total points and NEMS-S availability points while no significant correlation was found with NEMS-S total quality points and NEMS-S total price points. The lack of correlation of TP scores with NEM-S total quality scores in this study points to the value of measuring multiple variables in order to have a more comprehensive understanding of the food environment and socio-ecological drivers of food choice that influence dietary quality and health outcomes. Findings contribute to the body of literature that highlights that disparities exist in accessing healthy foods in the consumer food environment depending on rurality of location. Future research is needed to characterize food environments around the world based on total phenolic concentrations of FVs including in low-income, moderate-income, and high-income countries to evaluate if the findings found in this study are part of a larger trend. Additional research is further called for to examine if links exist between FV quality, food choices, dietary quality, and health outcomes. In addition, evidence-based food and nutrition interventions are called for to increase FV quality in rural consumer food environments where FV quality is relatively low by improving storage and distribution infrastructure. It is hoped that such research and management efforts will support access to high-quality FVs towards preventing diet-related chronic disease and reducing health disparities in consumer food environments.
